# Antibacterial and Antimycotic Activity of Cotton Fabrics, Impregnated with Silver and Binary Silver/Copper Nanoparticles

**DOI:** 10.1186/s11671-016-1240-0

**Published:** 2016-01-19

**Authors:** A. M. Eremenko, I. S. Petrik, N. P. Smirnova, A. V. Rudenko, Y. S. Marikvas

**Affiliations:** Chuiko Institute of Surface Chemistry of National Academy of Science of Ukraine, 17 General Naumov str., Kyiv, 03164 Ukraine; Institute of Urology of Academy of Medical Science of Ukraine, Yu Kotsyubynskogo, 9-A, Kyiv, 04053 Ukraine

**Keywords:** Bactericide cotton fabrics, Silver, Silver/copper nanoparticles, Antibacterial and antimycotic activity, 87.68.+z, 61.46.Df, 68.37.Ma

## Abstract

Effective method of obtaining of the bactericidal bandage materials by impregnation of cotton fabric by aqueous solutions of silver and copper salts followed by a certain regime of heat treatment is developed. The study of obtained materials by methods of optical spectroscopy, electron microscopy, and X-ray phase analysis showed the formation of crystalline silver nanoparticles (NPs) and bimetallic Ag/Cu composites with the corresponding surface plasmon resonance (SPR) bands in the absorption spectra. High antimicrobial and antimycotic properties of tissues with low concentrations of Ag and Ag/Cu nanoparticles (Ag/Cu NPs) (in the range 0.06–0.25 weight percent (wt%) for Ag and 0.015–0.13 wt% for Ag/Cu) is confirmed in experiments with a wide range of multidrug-resistant bacteria and fungi: *Escherichia coli*, *Enterobacter aerogenes*, *Proteus mirabilis*, *Klebsiella pneumoniae*, *Candida albicans* yeasts, and micromycetes*.* Textile materials with Ag NPs demonstrate high antibacterial activity, while fabrics doped with bimetallic composite Ag/Cu have pronounced antimycotic properties. Bactericidal and antifungal properties of the obtained materials do not change after a washing. Production of such materials is extremely fast, convenient, and cost-effective.

## Background

Silver and copper in the nanosize state are known for the antibacterial properties in relation to the wide spectrum of pathogenic and opportunistic bacteria. Last year in connection with development of steady strains of bacteria, their resistance to the antibiotics and bactericidal preparations is growing. Efficiency of silver as an antibacterial agent is known during centuries, and with the appearance of silver nanoparticles (Ag NPs), their use in different biomedical devices is growing sharply. Ag NPs in the colloid state have a large area of surface that results in the continuous release of Ag ions from the surface of Ag NPs and as a consequence to prolonged bactericidal effect. A few of reviews are devoted to the study of synthesis and antibacterial properties of silver NPs [1–3].

The mechanisms of silver NPs’ effect on a bacterial cell are discussed in the literature as the following: anchoring to the cell wall, accumulation of NPs, destruction of cell membrane by free radicals, interaction of silver with respiratory enzymes, release of reactive (singlet) oxygen, destruction of cell, and interaction with sulfur and phosphorus atoms of DNA [4]. A bactericidal action depends on the method of NP synthesis, their size and form, and also from the nature of stabilizer that protects NPs against oxidization and aggregation. In the case of NPs’ deposition on an inorganic or organic carrier, as a silica, polymer, colloid, or textile , the surface and chemical nature of carrier directly influence the release of active atoms and/or ions of metals and kinetics of their bactericidal action as well as the tendency to aggregate. Ag NPs are the original deposited form of silver ions, constantly generating and eliminating from the NP surface in the process of binding to biological substrates. Thus, locally (near the surface of the particles), generating high ion concentrations, is harmful to germs.

Herewith, the size of viruses is comparable with the sizes of cluster and colloid particles of silver. Ionizing potential of Ag NPs with the sizes of 1–2 nm is lower on 1.5 eV as compared to that of bulk silver, i.e., from the developed surface of NPs, the ions of silver are considerably generated easier. It provides more soft, prolonged action of preparations of cluster and colloid silver [5]. Unique properties have also copper nanoparticles (Cu NPs), which are less toxic, than ions of copper, and initial substances for a Cu NPs production is on a few orders cheaper than those that are used for the synthesis of Ag NPs. The free Cu^2+^ ions in high concentration can generate toxic effects, in particular due to the creation of reactive singlet oxygen, destroying amino acids and DNA [6]. Antibacterial activity of NPs is varied depending on the taxonomical location of microorganisms. For example, it was shown that Cu NPs have higher affinity to the amines and carboxyl groups on the surface of *Bacillus subtilis* than Ag NPs and therefore higher antibacterial activity [7]. The efficiency of Cu NPs synthesized in a chitosan [8] in relation to a *Collibacillus* is comparable with that of AgNPs. Cu NPs are easily oxidizable in air; at the same time, copper oxide demonstrates high enough bactericidal action [9–11]. In [12], the evolution of surface plasmon resonance (SPR) spectrum of Cu NPs in time is shown because of the formation of oxide layer and the decrease of the diameter of NPs’ core; herewith, the bactericidal action of the material remains high. Author [13] showed that the initial stage of damage of bacteria by different NPs (Ag, Hg, Cu) consists in the inhibition of cellular energy and structural changes of cellular surface. In [14], increased (as compared to their constituents) bactericidal ability of bimetallic nanoparticles (BMNP) Ag/Cu obtained by the method of chemical reduction in solution in the presence of stabilizers was shown; however, their stability was very low due to the separation into mono Ag and Cu particles and their oxidization. As stabilizers of NPs, surfactants, polymers, and amino acids are usually used. The last years a biogenic or “green” synthesis of metal NPs is popular with the use of bacteria, mushrooms, water plants, and plants. The advantage of biogenic methods is that NPs are reduced from ions and stabilized by the biomolecules produced by microorganisms that can be familiar to the human organism [15, 16]. In [17], to obtain Ag and Cu NPs, and also carbides and oxides of metals in air and in the atmosphere of nitrogen at temperatures from 160 to 600 °С, the suspensions of crystalline cellulose as a reducing agent has been used; a method is attributed also to the green synthesis. Previously, we reported photochemical and chemical syntheses of nanosized silver, gold, copper, and BMNP Ag/Au and Ag/Cu in the colloid state and on the surface of silica, saving high bactericidal activity during a few months [18]. BMNP Ag/Au possesses the expressed antitumoral action [19]. In accordance with XPS data [20], Ag/Cu nanoalloy in ultrathin polyelectrolyte films possesses high bactericidal activity. However, there is no doubt that antimicrobial drugs based on nanosized silver and copper have an irritating and toxic effect on the body. It can be assumed that the toxicity of NPs within the fabric is much less compared to that of other carriers due to the strong binding to the tissue structure, while maintaining availability for microorganisms. Thus, economically beneficial route to the creation of safe and effective biocidal materials with a simple method of preparation and storing a long time of application is an urgent task. This direction is of great scientific and applied interest. The very perspective is the introduction of NPs in woven fabrics for clinical application. Impregnation of Ag NPs in wool [21], cotton, covered by a hydrophobic polymer (norgine, rubbery anionic polysaccharide from red water plants) [22], viscose, nylon, and polyamide [23] have been presented. The impregnation of silver NPs in wool, polymeric, and cotton fabrics by the methods of plasma or thermal sputtering, electrochemical way [24, 25], laser ablation, and flaming synthesis [26–29] are presented. Ag NPs connect with the surface of the fabric in the form of crystallites and inhibit the expansion of polyresistant bacteria. The depth of penetration of NPs in a cotton is approximately 30 Å [30]. The thermal way of synthesis of Ag/Cu BMNP is comfortable from the point of view of lowering their melting temperatures; the alloy of Ag/Cu is formed without big energy expenses. Ag enhances oxidizing activity of copper, and BMNP Ag/Cu have higher surface reactivity as compared to their constituents [31]. In this work, the original method of impregnation of silver and bimetallic Ag/Cu NPs in bandaging cotton fabrics by their saturation with water solutions of silver and copper salts without application of chemical-reducing agents, at certain mode of heat treatment, not requiring substantial power inputs and special equipment is proposed.

## Methods

We use AgNO_3_ and CuSO_4_·5H_2_O from Aldrich. Gauze and madapollam fabrics were used as cotton samples. The surface of gauze is 36 g/m^2^ and that of madapollam is 94 g/m^2^.

### Production of Bactericidal Tissues Containing Ag NPs

Cotton textile was immersed in the water solutions of AgNO_3_ (1·10^−4^ ÷ 1·10^−1^ molar (M)) for 15 min then squeezed thoroughly and then evenly ironed at 200–220 °С by means of metallic press during 5 ÷ 10 min. Fabric is dyed in yellow or yellow-brownish color depending on the amount of silver on the unit of the fabric surface. A brownish tint appears because of the oxidization of part of silver to the oxide Ag_2_O. The concentration of silver on fabrics calculated from the adsorption isotherms of AgNO_3_ is 0.06–0.25 wt%. (60–250 percent per million (ppm))

### Production of Tissues Containing Copper

Cotton textile was saturated with water solutions of CuSO_4_ then squeezed thoroughly and evenly ironed at 200–220 °С by means of metallic press during 5 ÷ 10 min. Fabric acquires a greenish-brownish color depending on the amount of copper on the unit of the fabric surface. It should be noted that all samples of fabrics containing copper did not show the expressed bactericidal activity.

### Production of Bactericidal Tissues Containing BMNP Ag/Cu

Natural fabrics like gauze or madapollam were impregnated with mixture of water solutions AgNO_3_ and CuSO_4_ with ratio Ag:Cu = 1:1 within the fabric, then squeezed thoroughly and evenly ironed at 200–220 °С during 5–10 min. In fabrics with BMNP, ratio of Ag:Cu is equal within 0.015–0.13 wt% (15–70 ppm) depending on the concentration of initial impregnating solutions. These amounts were determined on the correlation of area under maximum absorption spectrum by Gaussian expansion. A calibration was performed as dependence of corresponding area and intensity of SPR spectrum on silver amount calculated from an adsorption isotherm. Fabric is dyed in a red-brownish color; the tint of that depends on the amount of the appearance of the metal on the unit of textile surface. Red-brownish tint appears because of the formation of protoxide and oxide of copper. The production of bactericidal fabrics by the ironed wet materials with the metal ions at a temperature near 200–220 °С does not require the use of chemical reductant and prolonged warming up; the surface of press is not painted and not corroded, allowing to save time and facilities at the production of material.

The results of action of fabrics impregnated with different concentrations of Ag NPs and Ag/Cu BMNP on bacteria, fungi of the genus *Candida*, and micromycetes are shown. We indicated the concentrations of initial salt solutions used for the impregnation of fabrics before their thermal treatment near each sample. Since the preparation conditions of the tissues, namely, the concentration of the salt solutions and the time of impregnation and drying of tissues were similar for all samples. Table 1 shows the concentration of the impregnation solutions near the symbol of each sample that will facilitate the reproduction of the results. Usually, a piece of cotton (10 g) was immersed in a 100 ml of solution of a certain concentration of silver, copper, or Ag/Cu salts for 30 min. Amounts of adsorbed salts were determined spectrophotometrically. Amounts of appearing Ag and BMNP Ag/Cu were determined on calibration bands of diffusion reflectance spectra (DRS) of dry fabrics after the ironing procedure.

**Table 1:** The results of action of fabrics impregnated with different concentrations of Ag NPs on bacteria, fungi of the genus Candida and mikromycetes

Test-culture			E.coli	K. pneumoniae	E. aerogenes	P. vulagaris	P. aeruginosa	S. aureus	E. faecalis	C. albicans	C. non-albicans	Rhodotorula glut.	Rhodotorula spp.	A.niger	A.flavus	Penicillium spp.	Alternaria alternata
nano-particles in colloid		Ag/glycerin	+	+	+	+	+	+	0	0	+	n/s	n/s	n/s	n/s	n/s	n/s
		Ag/SDS/PVP	+	+	+	+	+	+	0	+	+	n/s	n/s	n/s	n/s	n/s	n/s
non-particles on SiO_2_ powder		Ag	0	0	0	0	0	0	0	0	0	n/s	+	+	+	+	+
nonoparticiples on the cotton fabrics	Ag/ Cu (10·10^−3^)	gause	0	0	0	n/s	0	0	n/s	+	+	+	+	+	n/s	+	+
	Ag/Cu (1·10^−2^)		0	n/s	0	n/s	0	n/s	n/s	+	+	n/s	n/s	n/s	n/s	n/s	n/s
	Ag/Cu (1·10^−1^)		0	0	0	n/s	0	0	0/+	+	+	+	n/s	+	n/s	+	+
	Ag (1·10^−3^)	madapollam	+	+	0	+	+	+	0	+	+	n/s	+	+	0/+	+	+
	Ag (1·10^−2^)		0	0	0	0	0	0	0	0	0	n/s	+	+	0/+	+	+
	Ag (1·10^−1^)		0	0	0	0	0	0	0	0	0	n/s	+	+	0/+	+	+
	Ag (1·10^−3^)	gause	0	+	+	+	+	+	+	+	+	n/s	n/s	n/s	0/+	n/s	n/s
	Ag (1·10^−2^)		0	0	0	0	0	0	0	0	0	n/s	+	+	0/+	+	+
	Ag (1·10^−1^)		0	0	0	0	0	0	0	0	0	n/s	n/s	+	0/+	+	+
ions on the cotton fabrics	Ag+/Cu^2^+(1·10^−3^)	gause	+	+	+	n/s	+	+	n/s	+	+	n/s	n/s	n/s	n/s	n/s	n/s
	Ag+/Cu^2^+(1·10^−2^)		+	+	+	n/s	+	+	n/s	+	+	n/s	n/s	n/s	n/s	n/s	n/s
	Ag+/Cu^2^+(1·10^−1^)		+	+	+	n/s	+	+	+	+	+	+	n/s	+	n/s	+	+
	Cu^2^+(1·10^−3^)		+	+	+	n/s	+	+	n/s	+	+	n/s	n/s	+	n/s	n/s	n/s
	Cu^2^+(1·10^−2^)		+	0	0	n/s	+	0	n/s	+	+	n/s	n/s	+	n/s	n/s	n/s
	Cu^2^+(1·10^−1^)		0	+	0	n/s	0	0	0	+	+	+	n/s	+	n/s	+	+
	Ag+(1·10^−3^)		0	+	0	n/s	+	+	n/s	+	n/s	n/s	n/s	n/s	n/s	n/s	n/s
	Ag+(1·10^−2^)		0	0	0	n/s	0	0	n/s	+	n/s	n/s	n/s	n/s	n/s	n/s	n/s
	Ag+(1·10^−1^)		0	0	0	n/s	0	0	+	+	+	+	n/s	+	n/s	+	+

*0 - no growth of test cultures under samples*

*+ - Is the growth of test cultures*

*n/s - studies have not been conducted*

The DRS of the fabrics with NPs were registered by means of spectrophotometer PerkinElmer Lambda Bio UV-vis with the integrating sphere of Labsphere RSA-PR-20 in the range of waves 200–1000 nm. XRD analysis was performed by means of diffractometer DRON-407 with a nickel filter in the radiation of СuK_α_ (*λ* = 0.15418 nm) in the reflected bunch and registration geometry for Bregg-Brentano (2*θ* = 10°–60°).

To determine the effect of fabric materials impregnated with different concentrations of Ag and Ag/Cu nanoparticles on bacteria and micromycetes, we used a classic microbiological method. Petri dishes were filled with the respective test culture agar for bacteria and Sabouraud for fungi. Then, cotton fabrics were cut into equal-sized round pieces 10 mm × 10 mm, and the test cultures of bacteria (10^6^ CFU), yeasts of the genus *Candida* (10^5^ CFU), and the mold-forming fungi, micromycetes (10^5^ CFU) were placed on the cooled agar and carefully triturated with a spatula Drygalski on the surface of the agar. After drying up of inoculation, the tissue of investigated samples were applied onto the agar surface (1 cm × 1 cm) and Petri dishes were cultured in the conditions of thermostat: for bacteria at 37 °C for 24 h, for fungi of the genus *Candida* at 36 °C for 48 h, and for micromycetes at 28 °C for 3–5 days. The results of the action of fabrics impregnated with Ag NPs and Ag/Cu composites and the comparison of silver ions on the test cultures are presented in Table 1.

## Results and Discussion

**Fig. 1:**
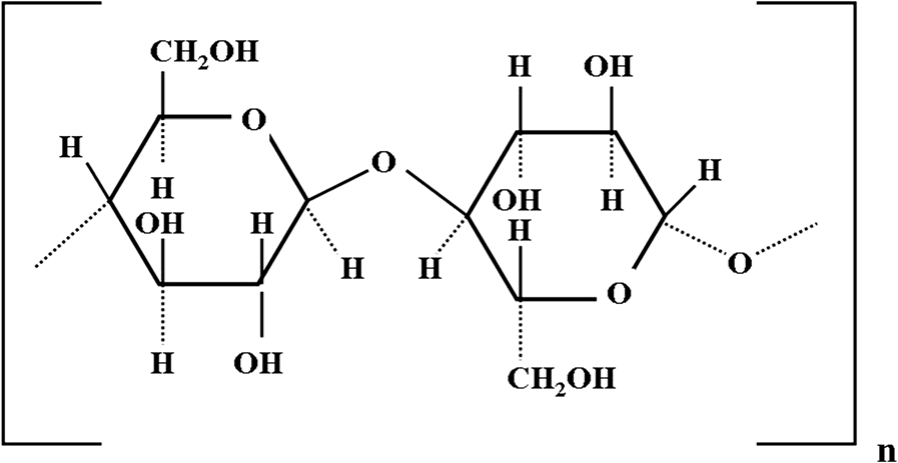
Structural formula of a fragment of the cellulose

The mechanism of reduction of metal ions to the NPs on the surface of the cotton by gentle heat treatment and their stability in relation to leaching of the metal particles or ions upon contact with water and biological fluids is not entirely clear. It is known that cotton is 99.6 % cellulose, and the rest is ash-like substance. Cellulose is a long chain polymer molecule consisting of repeating glucosidic residues, 300–10,000 glucose residues, without side loops. Сellulose contains reducing oligosaccharides. Their aldehyde function presumably can promote the process of silver and copper ion reduction (probably analogous to the reaction of “silver mirror”). Figure 1 shows a fragment of the polymeric chain of cellulose.

**Fig. 2:**
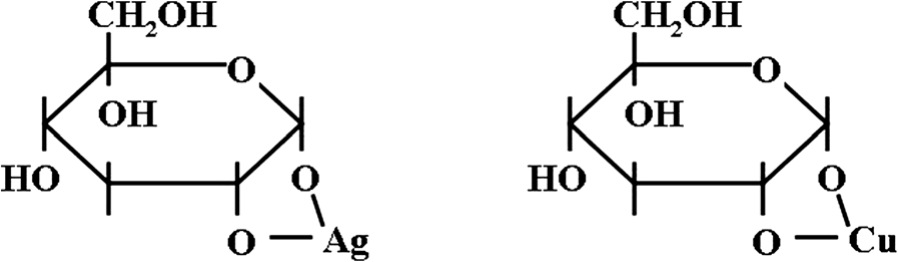
Formation a bond between the metal ion and OH group of cellulose

We can assume also the concentration of metal ions around OH- groups and the bond formation between the metal cations and hydroxyls of cellulose (Fig. 2). The water molecules are included in the coordination sphere of metal ions, simultaneously forming a hydrogen bond with OH- groups of cellulose. The Ag^+^ ions have a relatively high reduction potential and are reduced to Ag atoms at low temperature (160–200 °C in air). The reduction of copper ions requires a higher temperature; however, when impregnated in the cotton, there is a danger of destruction of tissue due to carbonization process. Therefore, the ironing of tissues soaked in a salt solution was carried out in all cases at temperatures of 200 to 220 °C. The OH- groups of cellulose are oxidized to carboxyl groups. The fabric retains its structure; herewith according to FTIR [17], stretching band at 1720 cm^−1^ decreases due to the interaction between carboxyl groups and metal ions.

**Fig. 3:**
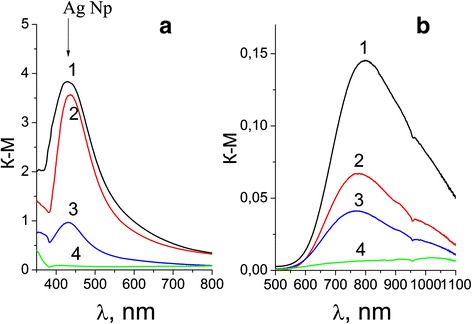
**a** Absorption spectra of tissues with Ag NPs corresponding to 1- 8 × 10^−2^ g/m^2^, 2- 8 × 10^−3^ g/m^2^, 3- 8 × 10^−4^ g/m^2^, and (4) - absorption spectrum of the original fabric. **b** Absorption spectra of tissues containing copper with the same concentration of Cu, g/m^2^ of tissue (4) - absorption spectrum of the original fabric

Figure 3 shows the electronic absorption spectra of cotton samples with different numbers of silver and copper particles after ironing, recalculated according to the equation of Kubelka-Munch. The presence of absorption band with a maximum at 430–440 nm (SPR) in Fig. 3a is indicative of the formation of silver NP_S_ in the structure of cotton.

**Fig. 4:**
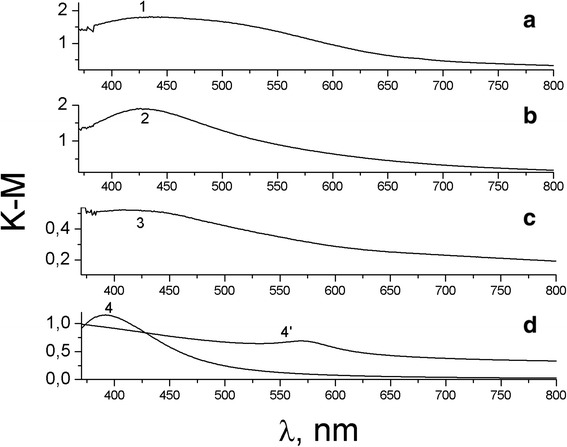
Absorption spectra of fabrics with BMNP (1–3); spectra 4- Ag NPs, C of BMNP with Ag: Cu ratio are 1- 8 × 10^−2^ g/m^2^, 2- 8 × 10^−3^ g/m^2^, 3- 8 × 10^−4^ g/m^2^; spectra 4- Ag NPs, 4’- Cu NPs in colloid solutions are shown for comparison

On the contrary, the wide absorption band in Fig. 3b does not correspond to the SPR spectrum of Cu NPs (max of SPR spectrum of Cu NPs is 565 nm) and rather belongs to the copper oxides on the fabric. The color of tissues changes from yellow to yellow-brownish for Ag and from greenish-brownish to brown for Cu depending on the metal amount. We assume that the impregnation of tissue with both silver and copper salts and subsequent heat treatment results in the reduction of the ions Ag^+^ and Cu^2+^ to nanoparticles of silver, yellow-brownish oxide Ag_2_O and a red-brown protoxide Cu_2_O. Metal particles remain in the structural micropores and on the surface of the fabric in the form of NPs or small aggregates. Formation of bimetallic particles Ag/Cu is complicated because of substantial distinction of oxidizing potentials—0.337 V for copper and 0.799 V for silver. Therefore, to control the process of simultaneous reduction of ions is difficult. In the absorption spectra of fabrics with BMNP on Fig. 4, a wide band with max near 750 nm is more typical for aggregates of Ag NPs. It is possible to suppose that in a bimetallic composites at heat treatment, Ag NPs precipitate on the copper oxide particles as a shell. As a whole, it is possible to suppose that under the impregnation and heat treatment of cotton with salts of metals, a basis of cotton, a cellulose, is simultaneously the reductant of ions and stabilizer of appearing NPs. This question needs further investigation.

In [19], the spectrum of Ag/Cu BMNP in a thin film of polyelectrolyte is attributed by the authors to the alloy also only slightly differs in the position of SPR of Ag NPs, though one would expect a much larger wavelength shift in the case of formation of the alloy. According to [19], in Ag/Cu alloy, silver and copper are close to each other in electrical contact and have a disordered random distribution of Ag and Cu atoms inside an enclosed structure and therefore do not possess crystallinity.

**Fig. 5:**
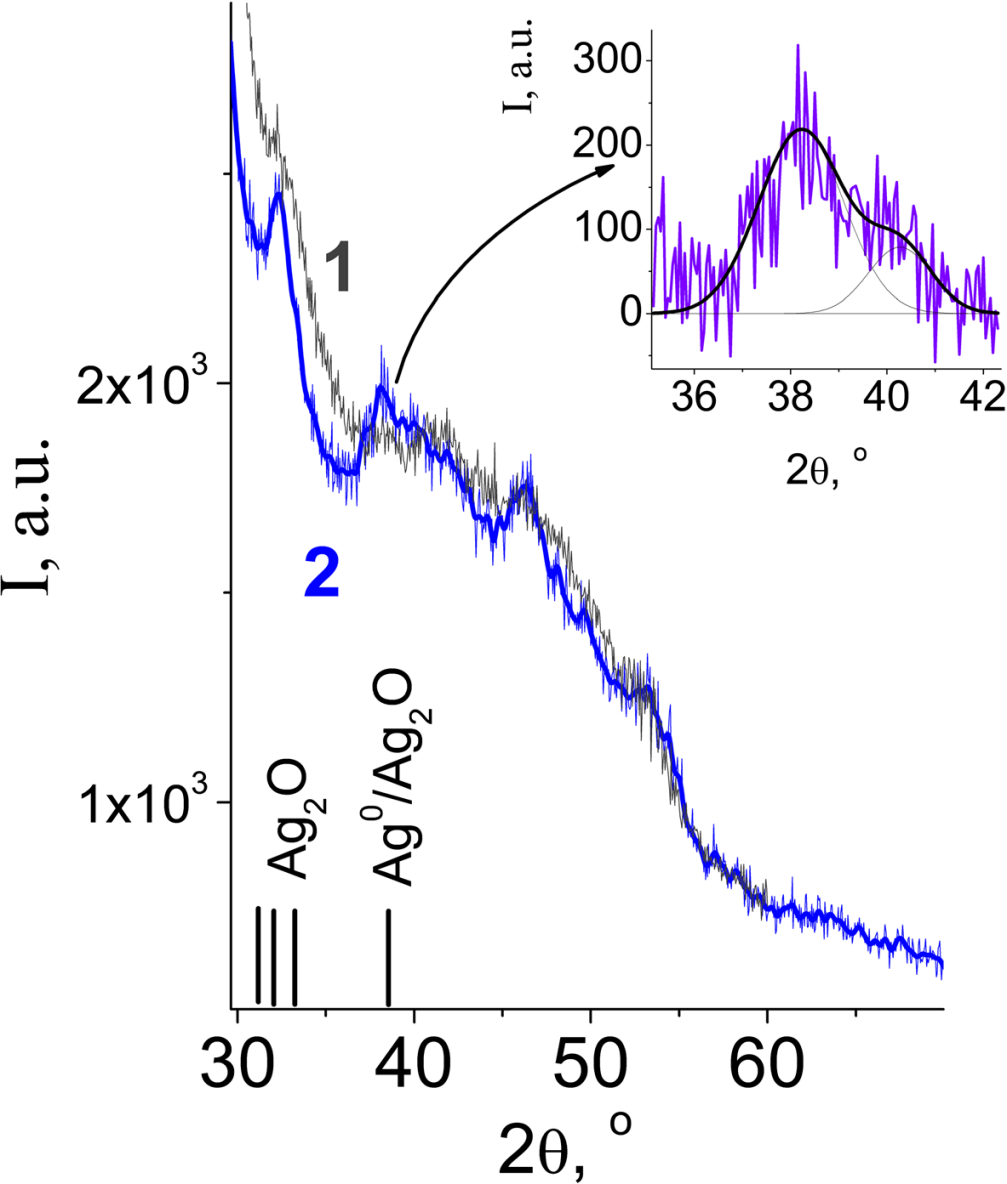
XRD of gauze tissue with impregnated Ag NPs

**Fig. 6:**
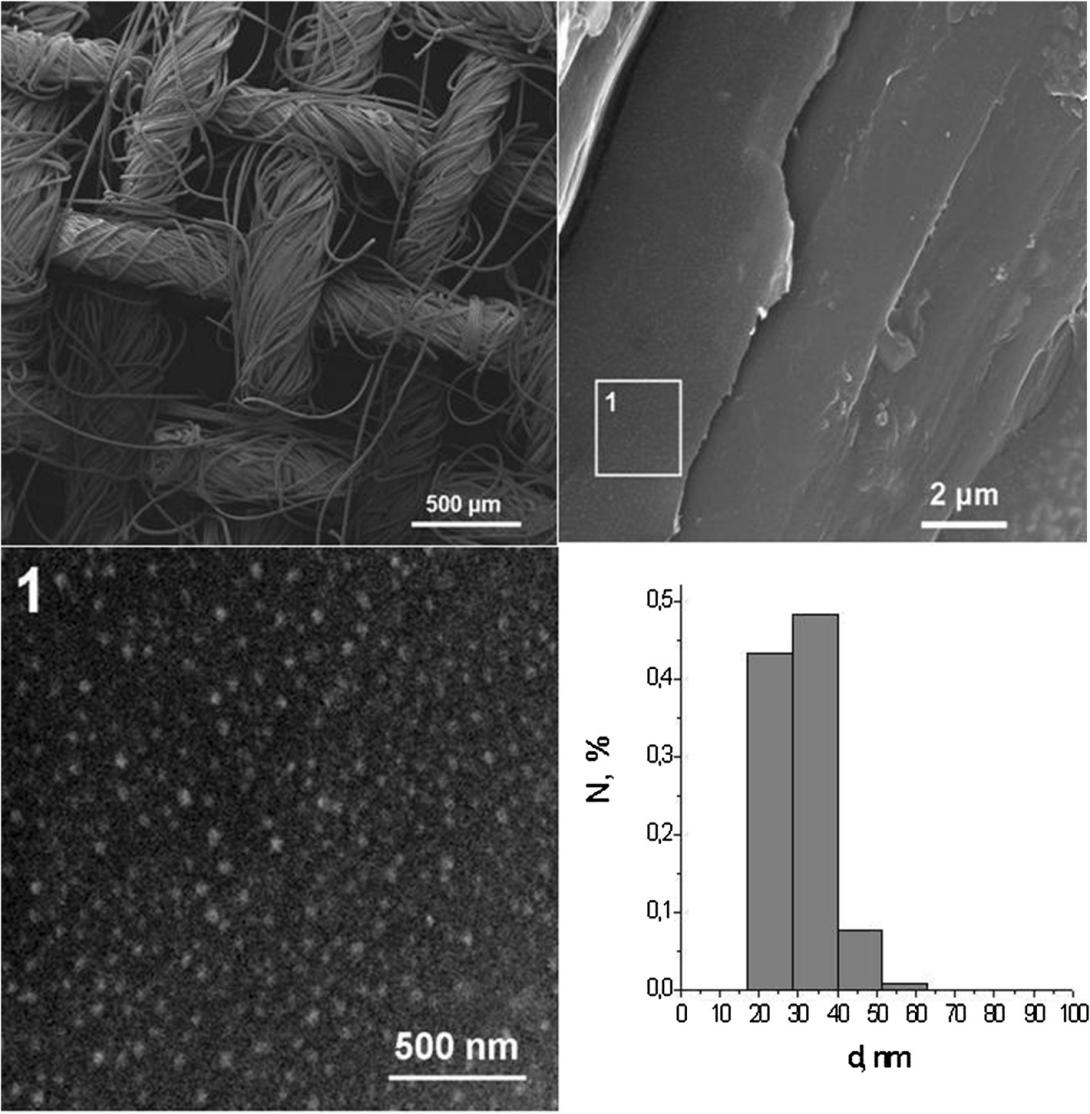
Electron microscopic image of the sample with BMNPs on gauze

Figure 5 shows the diffraction pattern of the tissue sample with silver NPs. Peak at 2*θ* = 32° is characteristic of silver oxide Ag_2_O and shows that the Ag NPs on the surface of the fabric are located in the shell of the silver oxide and a broad low-intensity band in the region of 2*θ* = 38° can be attributed to Ag^0^. Electron microscopic image of the BMNPs-impregnated gauze at different magnifications is shown in Fig. 6 (the scale bar is 500 μm on the left above, 2 μm on the right above, and 500 nm on the left down), as well as the size distribution of BMNP on the surface of the fabric. Ag/Cu NPs can be seen as white spherical spots which are uniformly disposed on the whole gauze surface. Average size of NPs is approximately 20–30 nm. Agglomeration of silver nanoparticles into larger clusters was also observed. The results showed that silver particles are sufficiently bound to the cotton fabric, which can retain good bacteriostatic properties even after washing.

Bactericide action of Ag NPs and BMNP are shown in Table 1. The samples of Ag-modified gauze and madapollam samples effectively kill the harmful bacteria and fungi. In the last two columns of Table 1, the results of bactericide activity of Ag NPs on the dispersive silica surface as well as of aqueous solution of colloidal silver obtained by us early are shown for comparison. Apparently, the activity of the obtained fabrics is comparable with such for Ag NPs in a colloid and on the developed surface of dispersive silica. Thus, advantages of fabrics are the substitution of noble metal silver on copper, simplicity of their production and storage, stability after washing, and maintenance of bactericidal action for a long time.

**Fig. 7:**
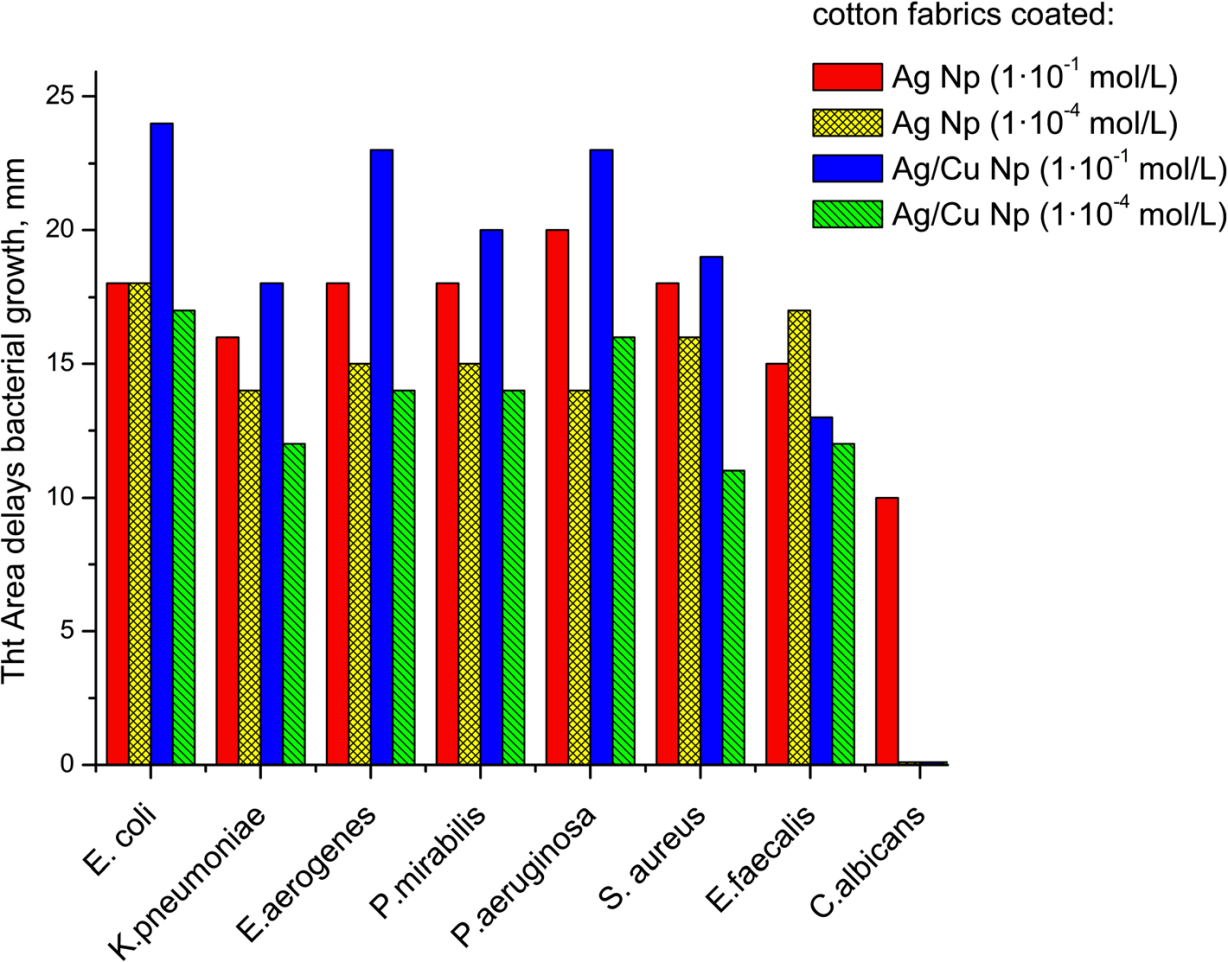
The effect of tissue samples with nanoparticles of silver and copper in clinical isolates of bacteria, fungi of the genus *Candida*, and micromycetes

The results of determining the effect of tissue samples with nanoparticles of silver and copper in clinical isolates of bacteria, fungi of the genus *Candida*, and micromycetes are shown in the diagram (Fig. 7)

.

Ratio Ag:Cu in BMNPs in gauzes is 1:1 (blue), with the content of metal 1.3·10^−5^:1.3·10^−1^ g/m^2^. Tissues with a corresponding number of binary Ag/Cu have pronounced bactericidal activity. As can be seen from the above data, the fabrics impregnated with BMNPs are the most effective in relation to most of the investigated test cultures. The maximal area of growth inhibition around the tissue for all studied bacteria, fungi, and micromycetes are detected for bimetallic composites. Cu ions and particles in the fabric do not found expressed antibacterial action. Kinetics of leaching of NPs with water from tissue samples were studied for 24 h. Metal ions were not detected in the solution, indicating the strong fixation of the particles on the fabric. Bactericidal activity is maintained for more than 6 months.

## Conclusions

Highly efficient bactericidal and antimycotic materials based on cotton fabrics contain nanoscale particles of silver and bimetallic Ag/Cu composition in an amount of 0.015–0.13 wt% obtained by impregnating a fabric with water solutions of corresponding metal salts followed by even ironing at 200 °C. The samples were characterized by optical spectroscopy, X-ray diffraction and electron microscopy and contain crystalline NPs of silver compounds with relevant SPR bands in the absorption spectra and bimetallic Ag/Cu composition—presumably the nanoparticles of copper oxide coated with silver NPs. High antimicrobial properties of tissues with Ag NPs and Ag/Cu composites are confirmed in experiments with a wide range of multidrug-resistant bacteria *Escherichia coli*, *Enterobacter aerogenes, Proteus mirabilis*, *K. pneumoniae*, *Candida albicans* yeasts, and micromycetes, and activity remains high throughout 6 months. Presumably under the impregnation of cotton with salts of metals and ironing at 200 °C, a basis of cotton, a cellulose, is simultaneously the reductant of ions and stabilizer of appearing NPs. Antibacterial fabrics do not reduce their activity after washing. A method developed for such material is extremely fast, cost-effective, and convenient.

## Abbreviations

A300: fumed silica
BMNP: bimetallic nanoparticles
M: molar
NPs: nanoparticles
wt%: weight percent
